# 

**Published:** 1897-12-11

**Authors:** 


**Notices of Births, Deaths, and Marriages** are inserted in this column at a charge of 2s. 6d. for an announcement not exceeding four lines.

### NOTICE TO CORRESPONDENTS.

All MS., Letters, Books for Review, and other matters intended for the Editor should be addressed The Editor, "The Hospital," The Hospital Building, 28 & 29, Southampton Street, Strand, W.C.

The Editor cannot undertake to return rejected MS., even when accompanied by stamped directed envelope.

**Notice to Advertisers and Subscribers.**

All Advertisements, Orders for Copies of The Hospital, and Business Communications should be addressed to **THE MANAGER, (Not to The Editor),** The Hospital, The Hospital Building, 28 & 29, Southampton Street, Strand, London, W.C.

The Cost of Subscription, post free, is as follows:—Per week, 2½d.; per quarter, 2s. 8d.; per half-year, 5s. 3d.; per annum, 10s. 6d. Foreign:—Per annum, 15s. 2d.; payable, in all cases, in advance.

### BOOKS RECEIVED.

H. K. Lewis.

"Mastoid Abscesses and their Treatment." By A. Broca, M.D,. and F. Lubet-Barbon, M.D. Translated and Edited by Henry J. Curtis, M.D.Lond., F.R.C.S.Eng.

Cassell and Co.

"The South African Climate." By William C. Scholtz, M.D.

Scientific Press.

"District Nursing on a Provident Basis." By Jamieson B. Hurry, M.A., M D.

Bennett Brothers, Bristol.

"Reports on Milk-Borne Enteric Fever." By D. S. Davies, M.D.

**Periodicals and Pamphlets Received.**—Scot's Pictorial, Literary Digest, Sunday at Home, Review of Reviews, Leisure Hour, Nursing Notes, Charity Organisation Review, Christmas Chimes (Christmas Number of the Girl's Own Paper), Boy's Own Paper, Boy's Own Paper Christmas Number, Boy's Sunday Monthly, The Scalpel, Monthly Supplement to Girl's Own Paper.

### THE STUDENT OF MEDICINE.

### APPOINTMENTS.

W. H. Bateman, M.B., Ch.B.Vict., appointed House Physician to the Manchester Royal Infirmary. H. E. Belcher, L.R.C.P.-Lond., M.R.C.S.Eng., appointed Medical Officer for the North and South Wilford, West Bridgford, and Gamston District of the Basford Union. Dr. Bennett, appointed Medical Officer for the Ruddington District of the Basford Union. Mr. M. G. Dyson, appointed Second Assistant Resident Medical Officer of the St. John's Road Workhouse of the Parish of Islington. C. H. Eccles, M.R.C.S.Eng., L.R.C.P.Lond., appointed Medical Officer for the Naverton District of the Driffield Union. F. Edge, M.D., F.R.C.S., M.R.C.P., B.Sc.Lond., appointed Surgeon to the Out-patients at the Birmingham and Midland Hospital for Women. J. Fletcher, M.B., C.M., D.P.H.Aberd., appointed Physician Superintendent of the Lightburn Joint Hospital for Infectious Diseases, Shettleston, Glasgow. G. E. Fryer, M.R.C.S., L.R.C.P., appointed Assistant Medical Officer to the Manchester Royal Infirmary. J. P. Lightfoot, M.R.C.S., L.R.C.P., appointed Medical Officer to the Bedale District of the Leyburn Union. S. Mullick, M.B., C.M.Edin., appointed Resident Medical Officer to the National Hospital for Diseases of the Heart and Paralysis, Soho Square, London, W. H. V. Palin, M.B., C.M.Edin., appointed Public Vaccinator for No. 1 District, Wrexham, and Divisional Surgeon, Police, Wrexham. F. J. Roberts-Dudley, M.R.C.S.Eng., L.S.A., appointed Certifying Factory Surgeon for the Borough of Stalybridge. Dr. Scott, appointed House Surgeon to the Teignmouth Hospital. G. H. Sheldon, M.B., Ch.B.Vict., appointed House Surgeon to the Manchester Royal Infirmary. N. J. Sinclair, M.B., Ch.B.Aberd., appointed House Physician and Surgeon to the Aberdeen Royal Hospital for Sick Children. H. C. Woodhouse, M.B., Ch.B.Vict., appointed House Surgeon to the Manchester Royal Infirmary.

St. Thomas's Hospital.—The following gentlemen have been selected as house officers from Tuesday, December 7th, 1897: House Physicians—H. E. Hewitt, L.R.C.P., M.R.C.S., W. McDougall, M.A., M.B., B.C.Camb. (extension), H. N. Goode, L.R.C.P., M.R.C.S. (extension), and H. C. Haslam, M.A., M.B., B.C.Camb., L.R.C.P., M.R.C.S.; House Surgeons—W. H. J. Paterson, L.R.C.P., M.R.C.S. (extension), A. W. Tuke, L.R.C.P., M.R.C.S. (extension), L. Gilbert, L.R.C.P., M.R.C.S. (extension), and S. N. Babington, L.R.C.P., M.R.C.S.; Assistant House Surgeons—J. F. McClean, L.R.C.P., M R.C.S. (extension), H. J. Marriage, L.R.C.P., M.R.C.S. (extension), J. S. Hall, L.R.C.P., M.R.C.S. (extension), and H. H. Sanguinetti, B.A., M.B., B.Ch.Oxon, L.R.C.P., M.R.C. (extension); Obstetric House Physicians—(senior) G. D. Hindley, B.A., Oxon, L.R.C.P., M.R.C.S., (junior) S. D. Turner, L.R.C.P., M.R.C.S.; Clinical Assistants in the Special Department for Diseases of the Throat—J. N. Walker, L.R.C.P., M.R.C.S., and A. D. Cowburn, L.R.C.P., M.R.C.S.; Clinical Assistants in the Special Department for Diseases of the Skin—E. Stainer, M.A., M.B., B.Ch.Oxon (extension), and E. H. Cobb, L.R.C.P., M.R.C.S. (extension); Clinical Assistants in the Special Department for Diseases of the Ear—H. T. M. Alford, L.R.C.P., M.R.C.S., and F. R. Martin, B.A.Camb., L.R.C.P., M.R.C.S.; Clinical Assistant in the Electrical Department—A. H. Gibbon, L.R.C.P.Edin., L.R.C.S.Edin.

